# Supplementary material for: Efficient cavity-mediated energy transfer between photosynthetic light harvesting complexes from strong to weak coupling regime
Source: Nat Commun. 2025 Jun 12;16:5300. doi: 10.1038/s41467-025-60616-5 (PMC12163064; doi:10.1038/s41467-025-60616-5)
Supplement: Supplementary file 1 — Supplementary Information [file 41467_2025_60616_MOESM1_ESM.pdf]

Supplementary Information

Efficient cavity-mediated energy transfer between photosynthetic light harvesting complexes from strong to weak coupling regime

Fan Wu[1]*, Tu C. Nguyen- Phan*[2]*, Richard Cogdell*[3]*, Tönu Pullerits*[1]**

[1] Division of Chemical Physics and NanoLund, Lund University, Sweden;

[2] School of Infection and Immunity, University of Glasgow, Glasgow, G128QQ, UK;

[3]School of Molecular Biosciences, University of Glasgow, Glasgow, G128QQ, UK

E-mail: tonu.pullerits@chemphys.lu.se

Contents

Supplementary Figure 1: Angle-resolved absorption (transverse electric mode) spectra of the high concentration LH2 containing microcavity sample.

Supplementary Note 1: Broadband pump probe spectra evolution of LH2 film and cavity samples.

Supplementary Figure 2: Broadband pump probe spectra of LH2 Film and cavity samples.

Supplementary Note 2: Calculation of the light intensity inside the microcavity

Supplementary Figure 3: Schematic of light path inside an FP microcavity and the reflection spectra of cavity mirrors.

Supplementary Note 3: Exponential fits of the pump probe kinetics.

Supplementary Note 4: Analysis of the non-specific photoexcitation induced effects in pump probe measurement.

Supplementary Figure 4: Calculated pump probe spectrum from all untargeted effects induced by pump excitation in LH2 cavity.

Supplementary Figure 5: Intensity dependent pump probe kinetics of low concentration LH2 film and weakly coupled LH2 cavity sample.

Supplementary Figure 6: Intensity dependent pump probe kinetics intermediately coupled LH2 containing cavity sample and the EEA results from all LH2 containing cavity samples.

Supplementary Figure 7: Intensity dependent pump probe kinetics R6G films and R6G containing cavity samples with pump excitation at 490 nm.

Supplementary Figure 8: Intensity dependent pump probe kinetics R6G films and R6G containing cavity samples with pump excitation at 550 nm.

Supplementary Figure 9: Steady-state transmission spectra and intensity dependent pump probe kinetics of strongly coupled R6G cavity samples with different cavity lengths.

Supplementary References

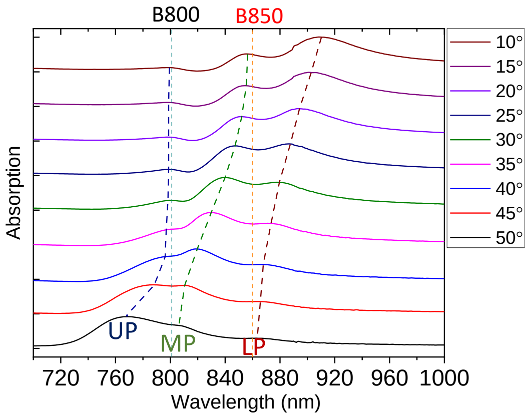

Supplementary Figure 1 Angle-resolved absorption (transverse electric mode) spectra of the high concentration LH2 containing microcavity sample.

Supplementary Note 1: Broadband pump probe spectra evolution of LH2 film and cavity samples.

The broadband pump probe spectra for all delay times of strongly and weakly coupled LH2 cavity samples, and high and low concentration LH2 films are plotted in Supplementary Figure 2. We can see that the pump probe spectra shapes for high concentration film, low concentration film and weakly coupled cavity sample are quite similar with obvious negative signals around 875 nm which corresponds to the GSB of B850 band and positive signals around 830 nm which corresponds the ESA of the B850 band. While the pump probe data of the strongly coupled LH2 cavity presented an obvious deviation from the other three samples. Here, we employed the rate-based kinetic model as proposed previously[1] to simulate the broadband pump probe spectra for all delays. We can see that the simulated model results agreed well with the experimental data for all the wavelength range, which validates this model and the corresponding energy relaxation pathway in the strongly coupled cavity system after the photoexcitation of the B800 band, i.e. B800 to B850_MP, B850_MP to DS, DS to B850_LP, B850_LP to DS, DS to ground states and B850_LP to ground states.

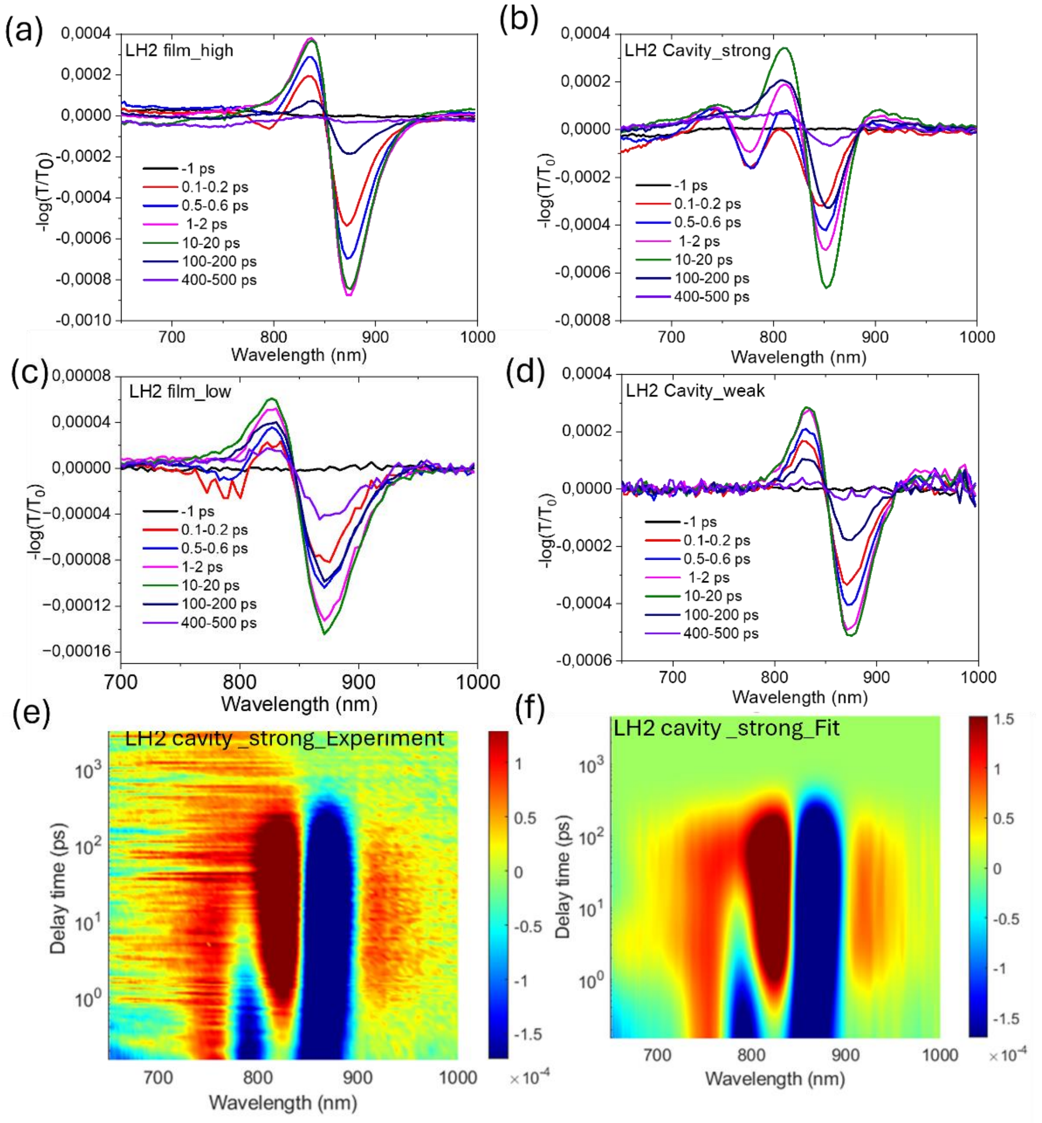

Supplementary Figure 2 Broadband pump probe spectra at all delay times of (a) high concentration LH2 film, (b) strongly coupled LH2 cavity, (c) low concentration LH2 film and (d) weakly coupled LH2 cavity sample, respectively; comparison of the (e) experimentally measured and (f) simulted pump probe spectra of the strongly couple LH2 cavity sample.

Supplementary Note 2: Calculation of the light intensity inside the microcavity

The pump excitation intensities inside and outside the cavity are compared here. Figure S1 shows the light path involving with an FP microcavity. The light intensity inside the cavity (I_{cav}) is estimated following the derivation in Chapter 3 of "Quantum Electronics for Atomic Physics" by Warren Nagourney[2]. Here, the total light intensity inside the cavity is calculated as the sum of the light intensity propagating towards the right and towards the left in the cavity. The the amplitude of the intracavity circulating field towards the right (E_{C_R}) is described as[2]:

$$E_{C\_R} = E_0 \frac{t_1}{1 - t^2 r_1 r_2 e^{-i\delta}} \quad (1)$$

Where E_0 is the amplitude of the incoming light field to the cavity, t_1 is the transmission amplitude coefficients of mirror 1, r_1, r_2 are the reflection coefficients of mirror 1 and 2, respectively, which are

identical in this work, t is the transmission amplitude coefficients of the absorptive film inside the cavity, δ is the phase shift as light traverses a round trip inside the cavity, which is defined as:

$$\delta = 2\pi \frac{2L}{\lambda_n} + 2\phi = 2\pi \frac{2L}{\lambda/n} + 2\phi = 2\pi \frac{2nL}{\lambda} + 2\phi \tag{2}$$

Where L is the length of the cavity, n is the refractive index of the film inside the cavity at the excitation wavelength λ, ϕ is the phase change after one mirror reflection, which equals π here.

Then the corresponding intensity travel towards right is:

$$I_{C\_R} = I_0 \left| \frac{t_1}{1 - t^2 r_1 r_2 e^{-i\delta}} \right|^2 \tag{3}$$

$$I_{C\_R} = I_0 \frac{T}{1 + t^4 r_1^2 r_2{}^2 - 2t^2 r_1 r_2 cos\delta} \tag{4}$$

$$I_{C\_R} = I_0 \frac{T}{1 + T_F{}^2 R^2 - 2T_F R cos\delta} \tag{5}$$

Where T is the transmission intensity coefficient of the mirror with $T=t_1^2$, R is the reflection intensity coefficient of the mirror with $R=r_1^2= r_2^2= r_1\, r_2$,considering mirror 1 and 2 are identical, T_F is the transmission intensity coefficient of the intracavity film with $T_F=t^2$, which can be obtained from the absorption spectrum of the corresponding bare film sample with $T_F= 10$^(-A), where A is the absorbance of the film at the excitation wavelength.

Similarly, the amplitude of the intracavity circulating field towards the left is:

$$E_{C\_L} = E_0 * t_1 * t * r_2 * e^{-i\theta} \tag{6}$$

$$+E_0 * t_1 * t * r_2 * t * r_1 * t * r_2 * e^{-3i\theta} \tag{7}$$

$$+ E_0 * t_1 * t * r_2 * t * r_1 * t * r_2 * t * r_1 * t * r_2 * e^{-5i\theta} \tag{8}$$

$$+ \cdots + E_0 * t_1 * t * r_2 * e^{-i\theta} * \left(t * r_1 * t * r_2 * e^{-2i\theta}\right)^n \tag{9}$$

$$E_{C\_L} = E_0 t_1 t r_2 e^{-i\theta} \sum_{n=0}^{\infty} \left[r_1 r_2 t^2 e^{-2i\theta}\right]^n \tag{10}$$

Where θ is the phase shift as light traverses a single trip inside the cavity with $\theta = \delta/2$. Since the terms within the sum above are all less than 1, we can use the math about geometric series:

$$\sum_{n=0}^{\infty} \left[r_1 r_2 t^2 e^{-2i\theta}\right]^n = \frac{1}{1 - t^2 r_1 r_2 e^{-2i\theta}} = \frac{1}{1 - t^2 r_1 r_2 e^{-i\delta}} \tag{11}$$

So:

$$E_{C\_L} = E_0 \frac{t_1 t r_2 e^{-i\theta}}{1 - t^2 r_1 r_2 e^{-i\delta}} \tag{12}$$

The corresponding intensity is:

$$I_{C\_L} = I_0 \frac{t_1{}^2 t^2 r_2{}^2}{1 + t^4 r_1^2 r_2{}^2 - 2 * t^2 r_1 r_2 cos\delta} \tag{13}$$

$$I_{C\_L} = I_0 \frac{TRT_F}{1 + {T_F}^2 R^2 - 2T_F R cos\delta} \quad (14)$$

Then the total intensity inside cavity is :

$$I_C = I_{C\_R} + I_{C\_L} = I_0 \frac{T + TRT_F}{1 + {T_F}^2 R^2 - 2T_F R cos\delta} = I_0 \frac{T + TRT_F}{1 + {T_F}^2 R^2 - 2T_F R cos\left(2\pi \frac{2nL}{\lambda}\right)} \quad (15)$$

For the LH2 containing cavities $T = 0.3, R = 0.7, L = 300nm, n = 1.48$, and T_F of the strongly coupled LH2 cavity $T_{F\_strong} = 0.91, T_F$ of the weakly coupled LH2 cavity $T_{F\_weak} = 0.96,$ which are obtained from the absorbances of the corresponding bare LH2 films. Thus, the intensity inside the strongly coupled cavity for pump excitation of 785nm is:

$I_{C\_LH2\_strong\_785nm} = 0.9I_0$.

The intensity inside the weakly coupled cavity for pump excitation of 800nm is:

$I_{C\_LH2\_weak\_800nm} = 1.1I_0$.

Thereout, we can conclude that the intensities inside and outside the LH2 cavities with these off-resonant pump wavelengths are roughly the same.

Similarly, the intensities inside the R6G cavities are calculated with pump wavelength at 490 nm (new experiments) and 550 nm, respectively.

When pump wavelength is 490 nm, $T = 0.2, R = 0.8, 2nL = 540nm, T_{F\_strong} = 0.84, T_{F\_weak} = 0.98$, then the intensity inside the strongly coupled cavity is:

$I_{C\_R6G\_strong\_490nm} = 0.9I_0$

And the intensity inside the weakly coupled cavity is:

$I_{C\_R6G\_weak\_490nm} = I_0$

When pump wavelength is 550nm, $T = 0.14, R = 0.86, 2nL = 540nm, T_{F\_strong} = 0.72, T_{F\_weak} = 0.96,$ the intensity inside the strongly coupled cavity is:

$I_{C\_R6G\_strong\_550nm} = 1.5I_0$

And the intensity inside the weakly coupled cavity is:

$I_{C\_R6G\_weak\_550nm} = 6I_0$

In summary, when the pump wavelength is far off resonant with the cavity mode, the light intensity inside the cavity is similar with or even smaller than the intensity outside the cavity. While when the pump wavelength is near resonant with the cavity, the intensity inside the cavity can be much larger than the intensity outside the cavity. This should be taken into consideration when evaluating the cavity induced effects.

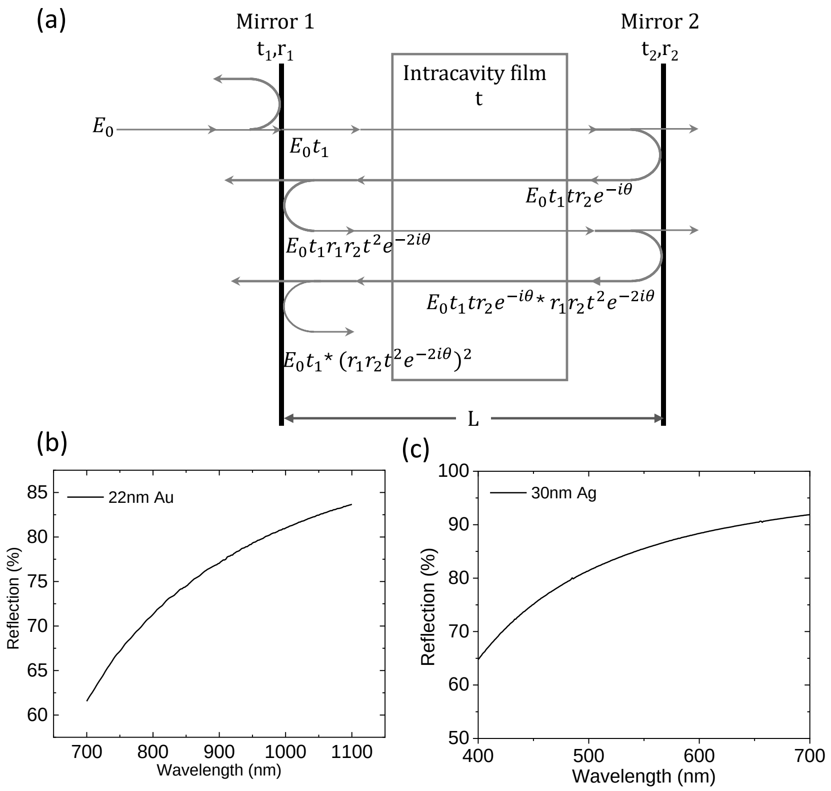

Supplementary Figure 3 (a) Schematic of light path inside an FP microcavity; (b) reflection spectrum of a 22 nm thick Au mirror on glass substrate;(c) reflection spectrum of a 30 nm thick Ag mirror on glass substrate.

Supplementary Note 3: Exponential fits of the pump probe kinetics

The intensity dependent pump probe kinteics of LH2 cavity and LH2 film samples are fitted with either single or two-exponential decays according to different samples. The pump probe kinetics of the strongly coupled LH2 cavity sample at different pump intensities are fitted with single exponential decay function $y = A * e^{-\frac{x}{t}} + y_0$, where the time constant t is the decay lifetime. While for high concentration LH2 film, to get good fit of the experimental data, the kinetics at different pump intensities are fitted with two-exponential function $y = A_1 * e^{-\frac{x}{t_1}} + A_2 * e^{-\frac{x}{t_2}} + y_0$, where the decay lifetime is then determined as the average of the two time constants t_1 and t_2 with $t_{average} = \frac{A_1 * t_1 + A_2 * t_2}{A_1 + A_2}$.

Supplementary Note 4: Analysis of the non-specific photoexcitation induced effects in pump probe measurement

The pump probe spectrum from all untargeted effects are calculated as reported previously[1]. In brief, we utilized the 3 by 3 coupled oscillator model as mentioned in the main text to calculate the microcavity optical properties. The input of the model included the absorption spectrum of the strongly coupled cavity sample at 30°, which was fitted using a series of Lorentzian functions. The parameters of the model are optimized by non-linear least squares method. Based on the model with optimized parameters, the transmission spectrum change with pump excitation induced all the non-specific effects, is computed as shown in Figure S4 which corresponds to a pump intensity of 8.5 μJ/pulse/cm^2 or 0.25 % bleaching. The non-specific polaritonic effects include the pump-induced thermal effect which leads to the thickness and refractive index change of LH2 film and the Rabi

contraction which was taken into consideration by decreasing the number of molecules participating in coupling induced by the pump excitation.

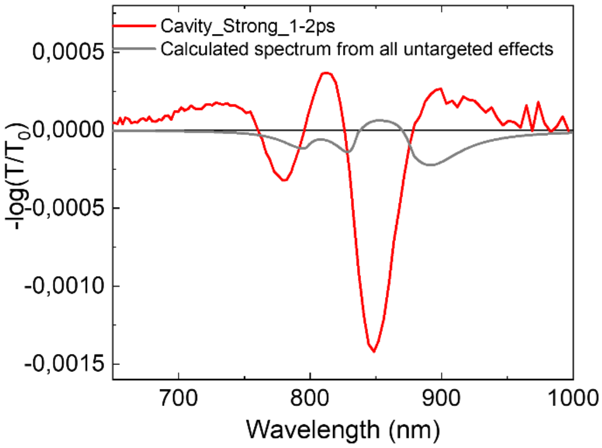

Supplementary Figure 4 Comparison of the calculated pump probe spectrum(grey) from all untargeted effects induced by pump excitation based on the coupled oscillator model [1]and the experimental pump probe spectrum (red) of the strongly coupled cavity sample.

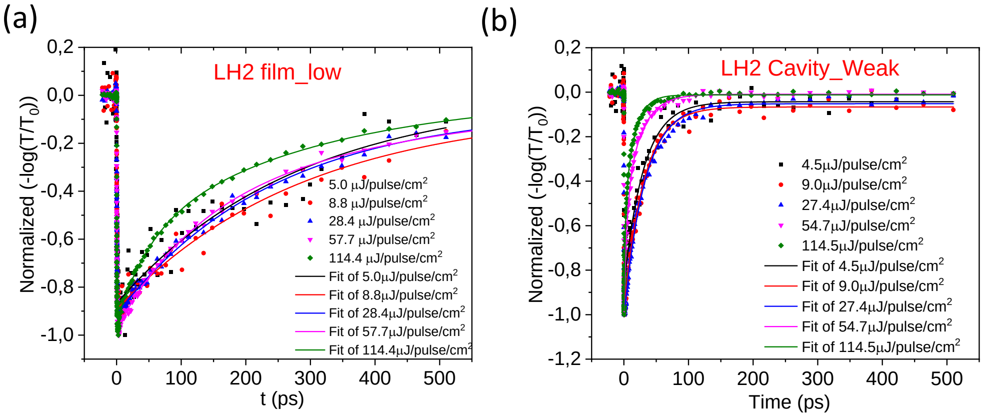

Supplementary Figure 5 Intensity dependent pump probe kinetics (scattered makers) with pump at 800 nm and probe at 875 nm of (a) low concentration bare LH2 film sample and (b) weakly coupled LH2 containing cavity sample. The solid lines are the fitting results based on exponential decays.

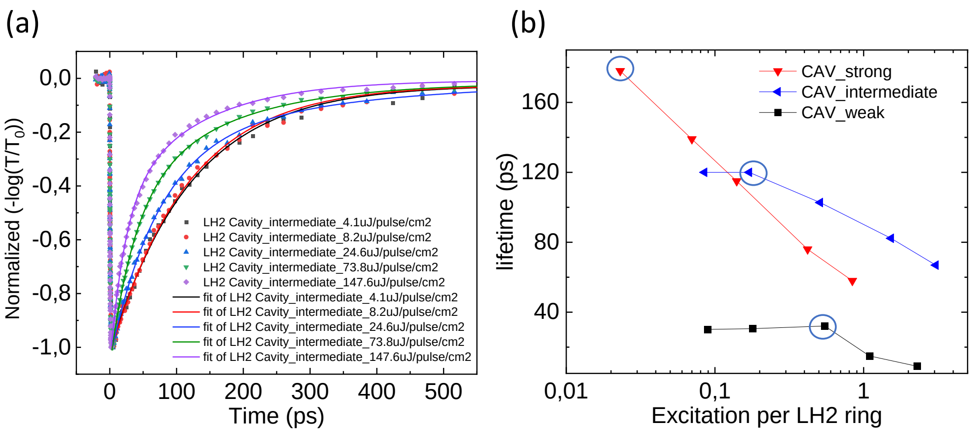

Supplementary Figure 6 (a) Intensity dependent pump probe kinetics (scattered makers) with pump at 800nm and probe at 875nm of intermediately coupled LH2 containing cavity sample (the solid lines are the fitting results based on exponential decays.); (b) the average lifetimes at different pump intensities fitted with exponential decays of strongly coupled LH2 cavity (red), intermediately coupled LH2 cavity (blue) and weakly coupled LH2 cavity sample (black).

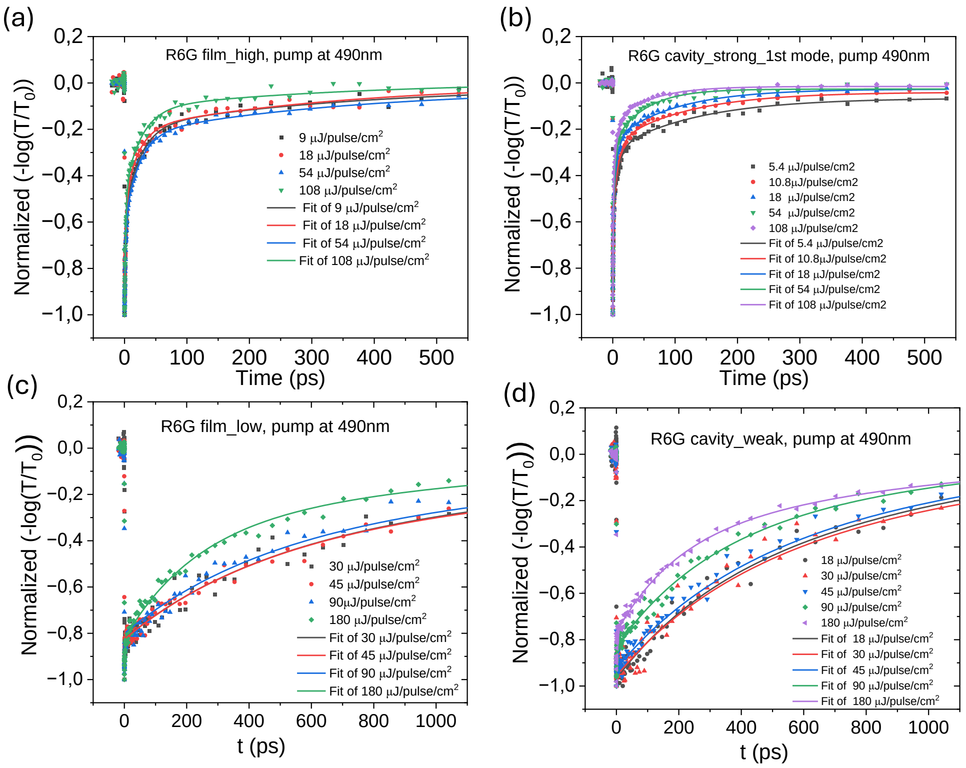

Supplementary Figure 7 Intensity dependent pump probe kinetics (scattered makers) with pump at 490 nm and probe at 560 nm of (a) high concentration bare R6G film sample and (b) strongly coupled R6G containing cavity sample and (c) low concentration bare R6G film sample and (d) weakly coupled R6G containing cavity sample, respectively. The solid lines are the fitting results based on exponential decays.

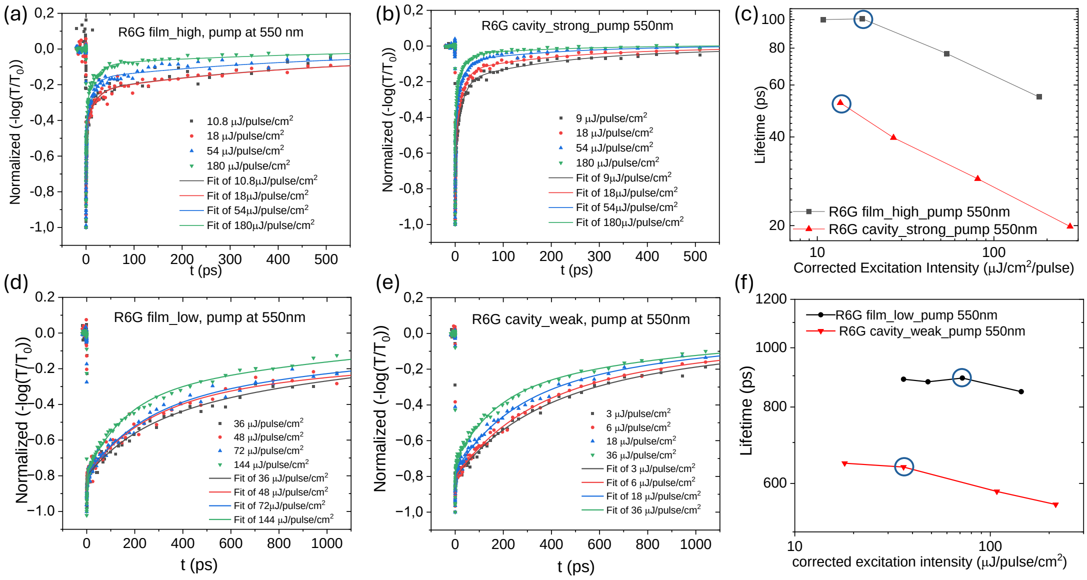

Supplementary Figure 8 Intensity dependent pump probe kinetics (scattered makers) with pump at 550 nm and probe at 560 nm of (a) high and (d) low concentration bare R6G film sample and (b) strongly and (e) weakly coupled R6G containing cavity sample (the solid are the exponential fits), respectively; the average lifetimes at different pump intensities obtained from exponential fit to experimental decays of (c) strongly coupled R6G containing cavity sample (red) and high concentration bare R6G film sample (black) and (f) weakly coupled R6G containing cavity sample (red) and low concentration bare R6G film sample (black).

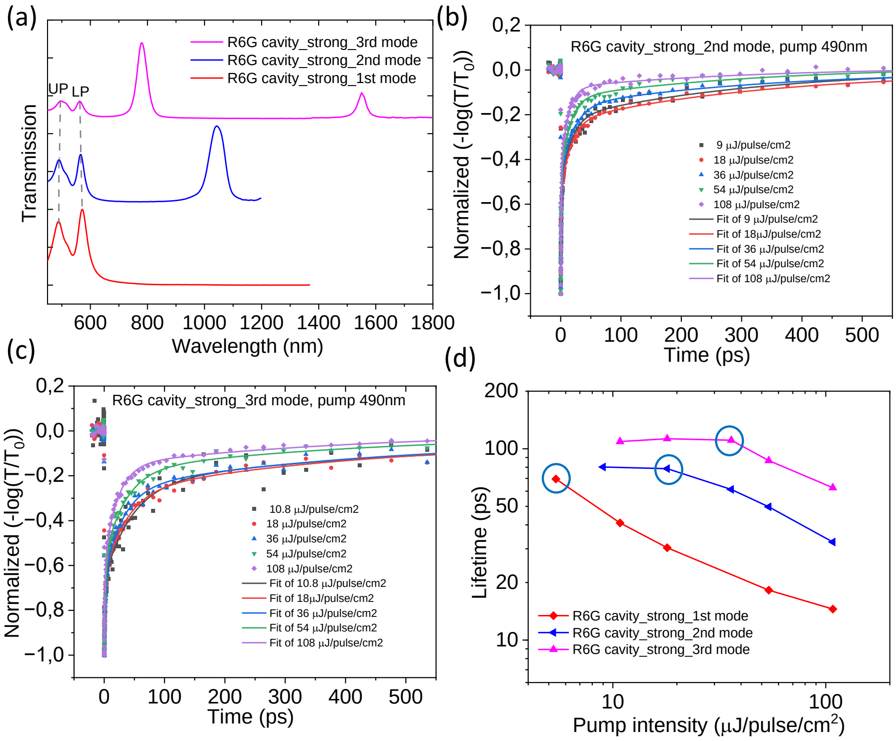

Supplementary Figure 9 (a) Steady-state transmission spectra of the R6G containing cavity samples where the first cavity mode (λ/2 cavity), second cavity mode (λ cavity) or third cavity mode (3λ/2 cavity) is strongly coupled with the exciton energy, respectively (dash lines indicates the upper polariton states-UP and lower polariton states-LP); Intensity dependent pump probe kinetics (scattered makers) with

pump at 490 nm and probe at 560 nm of the strongly coupled R6G cavity samples of the above mentioned (b) λ cavity and (c) 3λ/2 cavity sample (the solid are the exponential fits);(d) the average lifetimes at different pump intensities obtained from exponential fit to experimental decays of λ/2 (red), λ (blue), 3λ/2 (pink) strongly coupled R6G containing cavity samples.

Supplementary Reference

1. Wu, F. *et al.* Optical cavity-mediated exciton dynamics in photosynthetic light harvesting 2 complexes. *Nat. Commun.* **13**, 6864 (2022).
2. Nagourney, W. G. *Quantum Electronics for Atomic Physics and Telecommunication*. (Oxford University Press, 2014).
